# Deciphering the microbial communities in ticks of Inner Mongolia: ecological determinants and pathogen profiles

**DOI:** 10.1186/s13071-024-06512-1

**Published:** 2024-11-04

**Authors:** Chunfu Li, Rui Ma, Ai Gao, Na Jiang, Chunli Sang, Yanli Zhang, Haoqiang Tian, Jian Li, Wei Hu, Xinyu Feng

**Affiliations:** 1 School of Life Sciences, Inner Mongolia University, Hohhot, 010070 China; 2 Basic Medical College, Guangxi Traditional Chinese Medical University, Nanning, 530005 Guangxi China; 3 Department of Infectious Diseases, Huashan Hospital, State Key Laboratory of Genetic Engineering, Ministry of Education Key Laboratory for Biodiversity Science and Ecological Engineering, Ministry of Education Key Laboratory of Contemporary Anthropology, School of Life Sciences, Fudan University, Shanghai, 200438 China; 4 School of Global Health, Chinese Center for Tropical Diseases Research, Shanghai Jiao Tong University School of Medicine, Shanghai, 20025 China; 5 One Health Center, Shanghai Jiao Tong University-The University of Edinburgh, Shanghai, 20025 China

**Keywords:** *Ixodes persulcatus*, *Hyalomma asiaticum*, *Dermacentor nuttalli*, Tick microbiome, Feeding status, Geographical locations

## Abstract

**Background:**

Ticks are vectors of numerous pathogens, with their bacterial composition, abundance, diversity, and interaction influencing both their growth and disease transmission efficiency. Despite the abundance of ticks in Inner Mongolia, China, comprehensive data on their microbial communities are lacking. This study aims to analyze the microbial communities within ticks from Inner Mongolia to inform innovative control strategies for interrupting pathogen transmission.

**Methods:**

Tick samples were collected from animals and vegetation in multiple locations across Inner Mongolia and stored at − 80 °C. Ticks were identified using morphological keys and molecular biology methods. Full-length 16S rRNA gene sequencing was performed on collected samples. Bacterial community composition and diversity were mainly analyzed using bioinformatic tools such as QIIME, phyloseq, and DESeq2. Alpha diversity was assessed using Chao1, ACE, and Shannon indices, while beta diversity was evaluated using Bray-Curtis dissimilarity matrices. LEfSe analysis was applied to identify taxa associated with ecological and biological variables.

**Results:**

A total of 5,048,137 high-quality read counts were obtained, forming an average of 789.3 OTUs per sample. Proteobacteria, Firmicutes, and Bacteroidetes were the most dominant phyla. Bacterial community composition varied significantly with geography, with *Dermacentor nuttalli* showing a higher abundance of *Rickettsia* in Xilingol League, while other regions had different dominant genera. The microbial community also differed based on the feeding status of ticks. Additionally, the microbiota of engorged ticks showed organ specificity. Pathogen detection efforts revealed the presence of nine pathogens across all three tick species. *D. nuttalli* was found to carry a significantly higher burden of pathogenic bacteria, making it the most potentially threatening tick species in Inner Mongolia.

**Conclusions:**

The study highlights significant variations in tick microbiomes influenced by geographic location, feeding status, and tick species. It underscores the importance of enhancing tick and tick-borne disease surveillance in Inner Mongolia for early detection and control of emerging pathogens.

**Graphical Abstract:**

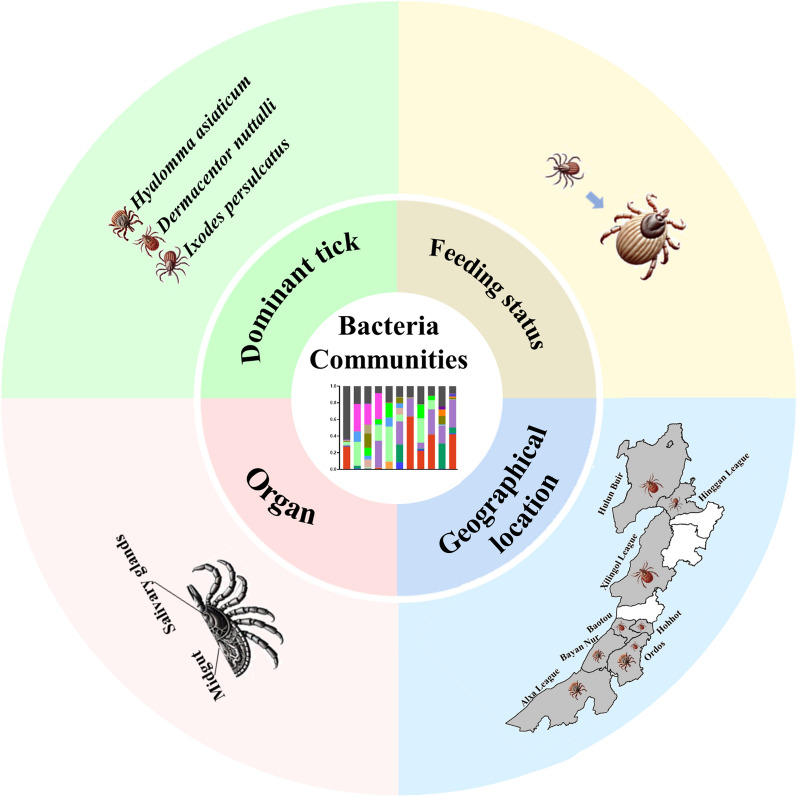

**Supplementary Information:**

The online version contains supplementary material available at 10.1186/s13071-024-06512-1.

## Background

Ticks are vectors for a wide array of zoonotic agents, affecting domestic and wild animals as well as humans [1]. These arthropods transmit various pathogens, including bacteria, viruses, fungi, and parasites, which posing significant public health risks [2]. Notable diseases spread by ticks include Lyme disease, tick-borne encephalitis, Crimean-Congo hemorrhagic fever, Q fever, tularemia, and North-Asian tick-borne spotted fever [3]. The incidence of tick-borne diseases is rising, driven by climate change and urbanization [4, 5]. Current control strategies are inadequate due to persistent tick populations, emerging acaricide resistance, and limited efficacy of personal protective measures. Innovative approaches, such as using microorganisms to control tick populations and interrupt pathogen transmission, are urgently needed [6–8].

The tick microbiome, consisting of symbiotic bacteria, is influenced by various factors, such as the tick's host and environmental conditions [9, 10]. Core microbiome genera, including *Coxiella*-like symbionts, *Rickettsia*, *Methylobacterium*, *Sphingomonas*, and *Francisella*-like symbionts, are consistently found across different tick species [11–15]. These microbes play crucial roles in tick biology, including pathogen susceptibility and transmission [16, 17]. Recent research has focused on understanding microbial communities' roles in pathogen transmission and developing novel control measures [18, 19]. Ticks acquire pathogens by feeding on infected hosts. Studies have shown that feeding increases bacterial richness in *Haemaphysalis longicornis* [20]. Pathogen infection can alter the tick microbiome, as seen in *Rhipicephalus microplus* and *Amblyomma maculatum* [21, 22]. The intra-tick microbiome interacts with internal organs and contributes to pathogen transmission, while the cuticular microbiome influences environmental survival and host interactions [23]. Additionally, bacterial community composition varies geographically, influenced by climate and vegetation [24–26].

The Inner Mongolia Autonomous Region, bordering Mongolia and Russia, is a critical area for tick-borne diseases in northern China [27]. The region's diverse flora and fauna and significant livestock populations make tick bites a serious threat, causing economic losses in animal husbandry [28]. Previous studies have documented the prevalence of 23 tick species in Inner Mongolia, including the dominance of species such as *D. nuttalli*, *Ixodes persulcatus*, *Dermacentor silvarum*, and *Haemaphysalis asiaticum*, and have identified a variety of tick-borne pathogens like the Yezo virus, tick-borne encephalitis virus, *Babesia*, *Theileria*, spotted fever group *Rickettsiae*, and *Coxiella burnetii* [29–33], several knowledge gaps remain that warrant further investigation. For instance, the bacterial community diversity, relative abundance, and composition within different tick species have not been thoroughly explored, which in turn affects the transmission potential of various pathogens. Additionally, the geographical variation in pathogen prevalence and tick species composition across different regions of Inner Mongolia needs more detailed data.

To better understand bacterial community diversity, relative abundance, and composition of tick species, we collected *I. persulcatus*, *H. asiaticum*, and *D. nuttalli* from various locations in Inner Mongolia. We analyzed microbial diversity, variation in bacterial community composition, and tick-borne pathogens using full-length 16S rRNA sequencing data. The findings will provide valuable insights for preventing and controlling tick-borne diseases in Inner Mongolia.

## Methods

### Tick collection and species identification

Tick samples were captured from animals (*Ovis aries*, *Camelus bactrianus*, *Capreolus pygargus*, *Equus caballus*) or the surface of vegetation in the Ordos, Baotou, Hohhot, Bayan Nur, Hinggan League, and Hulun Buir of Inner Mongolia and immediately stored at − 80 °C. Ticks were initially identified by morphological keys [35–37] and then confirmed by molecular biology methods using the mitochondrial cytochrome c oxidase subunit 1 gene (*cox1*) as previously described [38]. Briefly, ticks were first examined using a Nikon SMZ18 stereo microscope (Nikon). A fragment of the *cox1* was amplified by polymerase chain reaction for molecular identification (primers: TCOX1F: 5′- GGTCAACAAATCATAAAGATATTGG -3′ and TCOX1R: 5′-TAAACTTCAGGGTGACCAAAAAATCA-3′). Thermal cycling conditions were: a predenaturation step at 95 °C for 3 min, followed by 35 cycles of denaturation for 15 s at 95 °C, annealing for 15 s at 57 °C, and extension for 30 s at 72 °C, ending with 10 min at 72 °C.

### Sample collection and group classification

Over the past 2 years, we have collected a total of 2859 adult ticks representing three species from eight league cities across Inner Mongolia. For the subsequent microbiome analysis, 132 adult ticks were randomly selected. The ticks were subsequently categorized into groups based on four criteria: tick species, geographical location, feeding status and the dissected salivary glands and midguts. This categorization resulted in a total of 15 distinct groups, as detailed in Table 1.

**Table 1:** Sample grouping and collection sites

Tick species	Group	No. of samples (N)	Sample collection site
*D. nuttalli*	*D. nut*_Bao	7	Baotou
	*D. nut_*Ord	8	Ordos
	*D. nut_*Hoh	5	Hohhot
	*D. nut*_Xil	11	Xilingol League
	*D. nut*_Hul*_*UnE	12	Hulun Buir
	*D. nut*_Hul_En*g*	12	Hulun Buir
	*D. nut*_Hul_mid	12	Hulun Buir
	*D. nut_*Hul_sal	12	Hulun Buir
*H. asiaticum*	*H. asi*_Alx	7	Alxa League
	*H. asi*_Ord	14	Ordos
	*H. asi*_Bay	12	Bayan Nur
	*H. asi*_Bay_mid	12	Bayan Nur
	*H. asi_*Bay*_*sal	12	Bayan Nur
*I. persulcatus*	*Ixodes per*_Hin_En*g*	10	Hinggan League
	*I. per*_Hin_UnE	10	Hinggan League

"Eng" refers to ticks that have fed on the host. "UnE" refers to ticks that have not fed on the host. "mid" refers to the midgut of tick. "sal" refers to the salivary glands of tick. The salivary glands and midguts of 12 engorged *D. nuttalli* from Hulun Buir were dissected and analyzed, designated as *D. nut*_Hul_sal and *D. nut*_Hul_mid, respectively. Similarly, these tissues were dissected from 12 engorged *H. asiaticum* from Bayan Nur, designated as *H. asi*_Bay_sal and *H. asi*_Bay_mid

### DNA extraction

Each tick was washed three times with 70% ethanol to eliminate surface microbiological contaminants, though this procedure did not completely prevent DNA contamination originating from the tick cuticle and associated bacteria. The dissection of engorged ticks was conducted as previously described [39]. In brief, the dorsal cuticle was carefully removed, and specific organs, including the midgut and salivary glands, were extracted. These organs were then washed with sterile phosphate-buffered saline (PBS, 1X) and stored in 70% ethanol. The cleaned ticks or isolated tick organs were homogenized using a mechanical homogenizer in centrifuge tubes containing 500 μl of chilled PBS. The homogenized samples were subsequently centrifuged at 4 °C and 12,000*g* for 10 min, after which the supernatant was collected. Total DNA was extracted from the supernatant using the QIAamp DNA Mini Kit (Qiagen) according to the manufacturer's protocol. The purity of the extracted DNA was assessed via agarose gel electrophoresis, and the DNA samples were stored at − 80 °C until further use.

### Full-length 16S rRNA gene sequencing

DNA extracted from individual ticks or tissues was used for full-length 16S rRNA gene sequencing, facilitating the identification and differentiation of bacterial species. Sequencing libraries were prepared by amplifying full-length 16S rDNA using Barcode's specific primers and Phusion® High-Fidelity PCR Master Mix with GC Buffer (New England Biolabs) as previously described [40]. Amplified DNA fragments were purified using a PCR purification kit (Qiagen), and the sequencing adapters were ligated to both ends using the DNA adhesive enzyme. The excess sequencing adapters were removed using AMpure PB beads (PacBio). Then, DNA fragments of a specific size were selected through the BluePippin system (Sage Science). The DNA fragments were purified again using AMpure PB beads (PacBio), and library construction was completed using the SMRT bell Template Prep Kit (PacBio). The DNA fragments were purified again using AMpure PB beads (PacBio) and, after determining the concentration and the size of the insert of the constructed library, sequenced on the Pacific Biosciences (PacBio) platform according to the standard protocols.

### Sequencing data processing

PacBio sequencing data were processed using CCS (SMRT Link v7.0); the minimum number of passes in the sequence was three, and the minimum predicted accuracy was 0.99. The reads with a sequence length < 1340 bp or > 1640 bp were discarded. The reads were assigned to samples based on their unique barcode, and the barcode and primer sequence were cut off using cutadapt (version 4.1). Chimeric sequences are detected by comparing reads with a reference database using the UCHIME algorithm and then removing chimeric sequences [41, 42]. The valid data (Clean Reads) were finally obtained and saved in fastq format. Operational taxonomic unit (OTU) clustering was done with Uparse software (Uparse v7.0.1001), using a 97% identity cutoff to represent one species per OTU. According to its algorithm principle, the highest frequency sequence in each OTU’s cluster was used as the representative sequence. Taxonomic assignment of each cluster was carried out using Mothur (version 1.35.1) to match a representative sequence in each OTU to sequences from the SSUrRNA database of SILVA (version 138.1). Taxonomic information on bacteria from phylum to species was obtained in all samples.

### Bacterial community composition, relative abundance, and diversity analyses

In our study, we performed a comprehensive analysis of bacterial community composition and diversity, employing several advanced bioinformatic tools to ensure accurate data interpretation. Initially, OTU abundance information was normalized using the standard of the minimum sequence number across samples, employing the feature-table plugin of QIIME 2 software suite (version 2021.4). This normalization process was essential for subsequent analyses by mitigating the impact of sample size variability on our findings. Additionally, the relative abundance of microbial taxa was quantified and analyzed using the phyloseq package in R (version 1.36.0), which integrates phylogenetic, taxonomic, and abundance data, offering a robust framework for ecological analysis of microbiome data. To identify significant differences in the bacterial community composition associated with factors such as tick species, geographic location, and feeding status, we conducted differential abundance analysis using the DESeq2 package (version 1.30.1).

For alpha diversity analysis, we employed multiple indices calculated using QIIME (version 1.9.1), which provided insights into the richness and evenness of microbial communities. Richness was assessed using the Chao1 and ACE indices, which estimate the total number of distinct species (operational taxonomic units, OTUs) within the community. Evenness was measured using the Shannon index, which reflects the uniformity of species distribution within the samples. Phylogenetic diversity was evaluated with the Phylogenetic Diversity Whole Tree Index, capturing the breadth of phylogenetic relationships among the species present. Sampling completeness was assessed using Good's coverage index. For statistical comparisons of alpha diversity between different levels of variables, we initially used Kruskal-Wallis test and Wilcoxon rank-sum tests. However, to address concerns about multiple comparisons, we have now applied the Bonferroni correction to adjust the significance levels accordingly. Rarefaction curves were drawn using R software (version 2.15.3) to represent these data and evaluate sample sufficiency visually.

For beta diversity analysis, we calculated Bray-Curtis dissimilarity matrices to evaluate differences between the microbial communities of our samples. The results were visualized through Principal Coordinates Analysis (PCoA) and further analyzed using the unweighted pair-group method with arithmetic means (UPGMA) to explore the clustering patterns of microbial communities. Additionally, ANOSIM analysis, utilizing the adonis function of the R vegan package, was applied to test the statistical significance of observed patterns in microbial community structure across different groups.

Lastly, to highlight the taxa most likely to explain differences between groups, Linear Discriminant Analysis Effect Size (LEfSe) was performed using LEfSe software, with an LDA score filter value of 4. This method enabled us to pinpoint specific microbial taxa that are significantly associated with the ecological and biological variables in our study. Statistical comparisons between groups were executed using the t-test or Wilcoxon rank sum test, with a significance threshold set at *P* < 0.05.

### Tick-borne pathogenic bacteria

Taxonomic classification of pathogenic bacteria in humans was performed as previously described [43]. Briefly, based on the full-length sequences of the 16S rRNA gene obtained, the 16SPIP (version 0.1.1) program was utilized to BLAST against the 16S pathogen database by employing the sensitive mode. The 16S pathogen database comprises 159 bacterial species relevant to human health, as listed in “The Directory of Pathogenic Microorganisms Infecting Humans,” published by the Ministry of Health of the People’s Republic of China (MOHC). Importantly, the threshold of > 99% identical to the reference sequence ensures a high degree of confidence in the species assignments. This threshold reflects the close relationship required for accurate identification. Furthermore, we also cross-referenced our findings with existing literature reports and databases on tick-borne pathogens to ensure the accuracy of our identification.

## Results

### Sequencing data statistics

A total of 156 full-length 16S rRNA gene sequencing libraries were built based on tick species, sampling locations, and organs. Sequencing these samples yielded 5,548,746 raw read counts. After a filter procedure, 5,045,137 high-quality read counts that accounted for nearly 91% of the total read counts were used for data analysis. The number of obtained read counts varied among samples, ranging from 12,010 to 66,203, with average read counts of 32,317 per sample. The median length of the reads was 1461 bases. Based on a 97% similarity cutoff, these reads were formed into OTUs, with an average of 789.3 OTUs found per sample. The rarefaction curves generated from these samples' OTUs approached saturation, indicating that the sequencing coverage was sufficient for further analysis. A good coverage value for each sample (> 0.99) indicated the sequencing depth was sufficient to reveal most of the microbial community of samples.

### General bacterial community composition and relative abundance

**Fig. 1:**
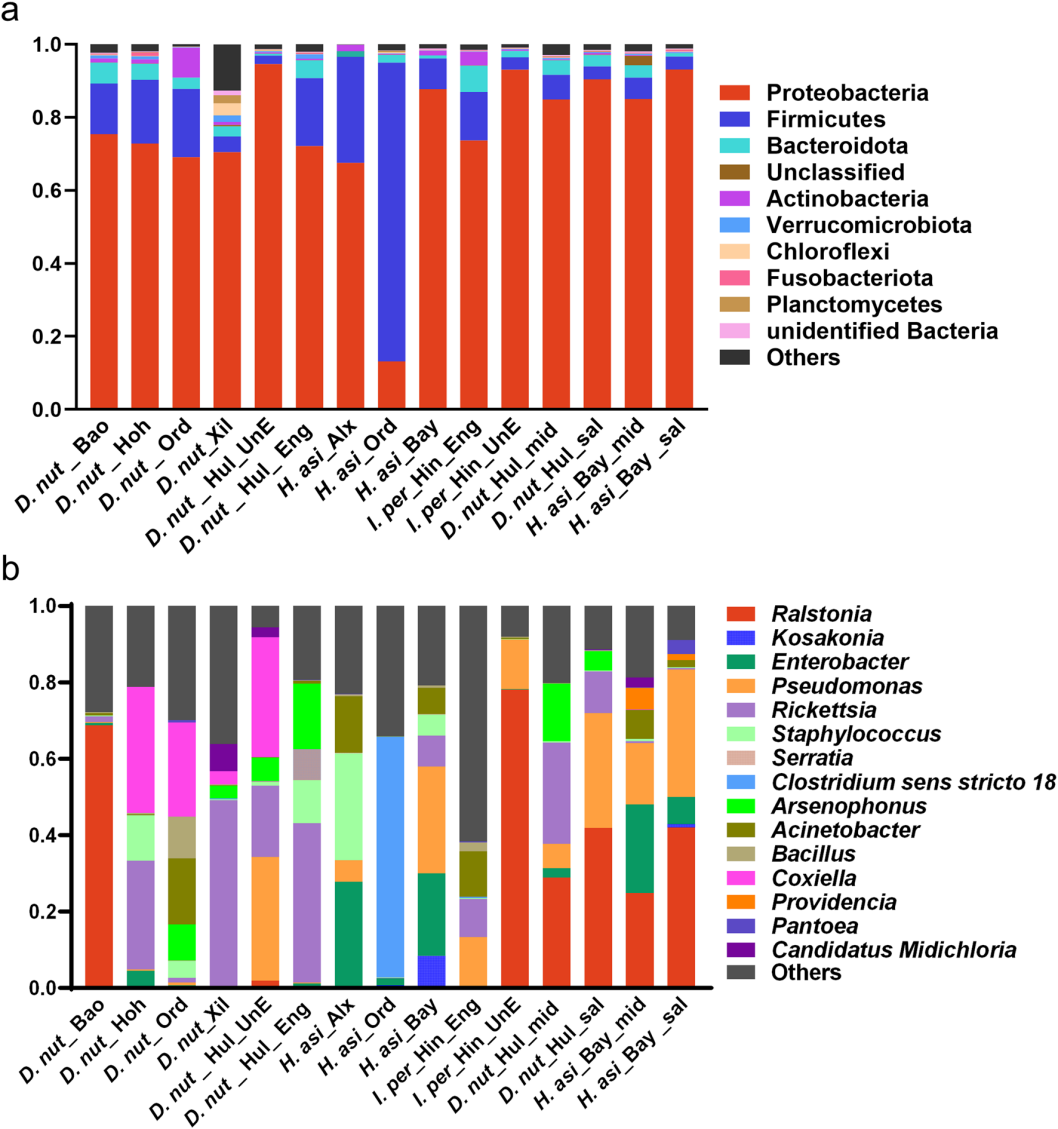
Relative abundance of bacterial community compositions at phylum (**a**) or genus (**b**) levels

All the OTUs were assigned to 142 phyla, 278 classes, 503 orders, 752 families, 1661 genera, and 2708 species. At the phylum level, 15 groups exhibited similar bacterial communities. The top 10 abundant phyla were Proteobacteria, Firmicutes, Bacteroidetes, Unclassified, Actinobacteria, Verrucomicrobia, Chloroflexi, Fusobacteria, Planctomycetes, and unidentified Bacteria. Proteobacteria was the most dominant phylum in all samples, followed by Firmicutes and Bacteroidetes (Fig. 1a, Tables S1, S2). However, there were significant differences at the taxonomic genus level (Fig. 1b, Tables S1, S2). In addition, we also identified many bacteria at the species level, among which *Ralstonia pickettii*, *Atlantibacter hermannii*, *Pseudomonas azotoformans*, *Staphylococcus xylosus*, *Citrobacter portucalensis, Acinetobacter guillouiae*, *Staphylococcus hominis*, *Escherichia coli*, and *Lactobacillus murinus* had high relative abundance in all samples (Tables S1, S2).

The bacterial community composition at the phylum level was similar among *D. nuttalli*, *H. asiaticum*, and *I. persulcatus* (Fig. 1a). At the genus level, *I. persulcatus* was dominated by *Ralstonia* (39.1%), while *H. asiaticum* showed higher relative abundances of *Enterobacter* (17.1%)*, Pseudomonas* (11.2%), and *Clostridium *sensu stricto* 18* (21.0%) (Fig. 1b). In contrast, *D. nuttalli* had higher relative abundances of *Rickettsia* (23.4%) and *Coxiella* (15.4%) (Fig. S1a).

In terms of geographic locations, *D. nuttalli* from Xilingol League exhibited a significantly higher abundance of *Rickettsia* than other geographical locations. *D. nuttalli* from Baotou displayed a higher relative abundance of *Ralstonia*, *D. nuttalli* from Ordos exhibited a higher relative abundance of *Bacillus*, *D. nuttalli* from Hohhot showed a higher abundance of *Coxiella*, and *D. nuttalli* from Hulun Buir had a higher relative abundance of *Pseudomonas*. In addition, the relative abundance of bacteria in *H. asiaticum* from Alxa League, Ordos, and Bayan Nur also exhibited differences. Compared with Alxa League and Bayan Nur, *H. asiaticum* from Ordos contained the highest relative abundance of *Candidatus Midichloria* (Fig. 1b, Fig. S1b, c, Table S1).

Comparative analysis of the midgut and salivary glands in *D. nuttalli* and *H. asiaticum* revealed significant variations between these organs in terms of microbial abundance. Specifically, the *Ralstonia*, *Pseudomonas*, and Burkholderiaceae were significantly more abundant in the salivary glands compared to the midgut in both *D. nuttalli* and *H. asiaticum* (*p* < 0.05, Fig. S1e, Table S1). In comparison to the midgut of *D. nuttalli*, the midgut of *H. asiaticum* exhibited a higher relative abundance of *Enterobacter*, *Acinetobacter*, and *Providencia* while a lower abundance of *Ralstonia*, *Rickettsia*, and *Mycoplasma* (*P* < 0.05). Additionally, distinct microbiomes were identified in the midguts of the two tick species; *Rickettsia* predominated in *D. nuttalli*, whereas *Mycoplasma* was more prevalent in *H. asiaticum*.

Significant difference in bacterial community composition were observed between *D. nuttalli* and *I. persulcatus* in different feeding states. Notably, *Arsenophonus*, *Rickettsia*, and *Staphylococcus* were more abundant in engorged *D. nuttalli*, while *Pseudomonas* and *Coxiella* predominated in unengorged *D. nuttalli*. Similarly, *Rickettsia* and *Acinetobacter* were more abundant in unengorged *I. persulcatus*, with *Ralstonia* having higher relative abundance in unengorged *I. persulcatus* (Fig. S1d, Table S1).

### Microbial variation among *D. nuttalli*, *H. asiaticum*, and *I. persulcatus*

**Fig. 2:**
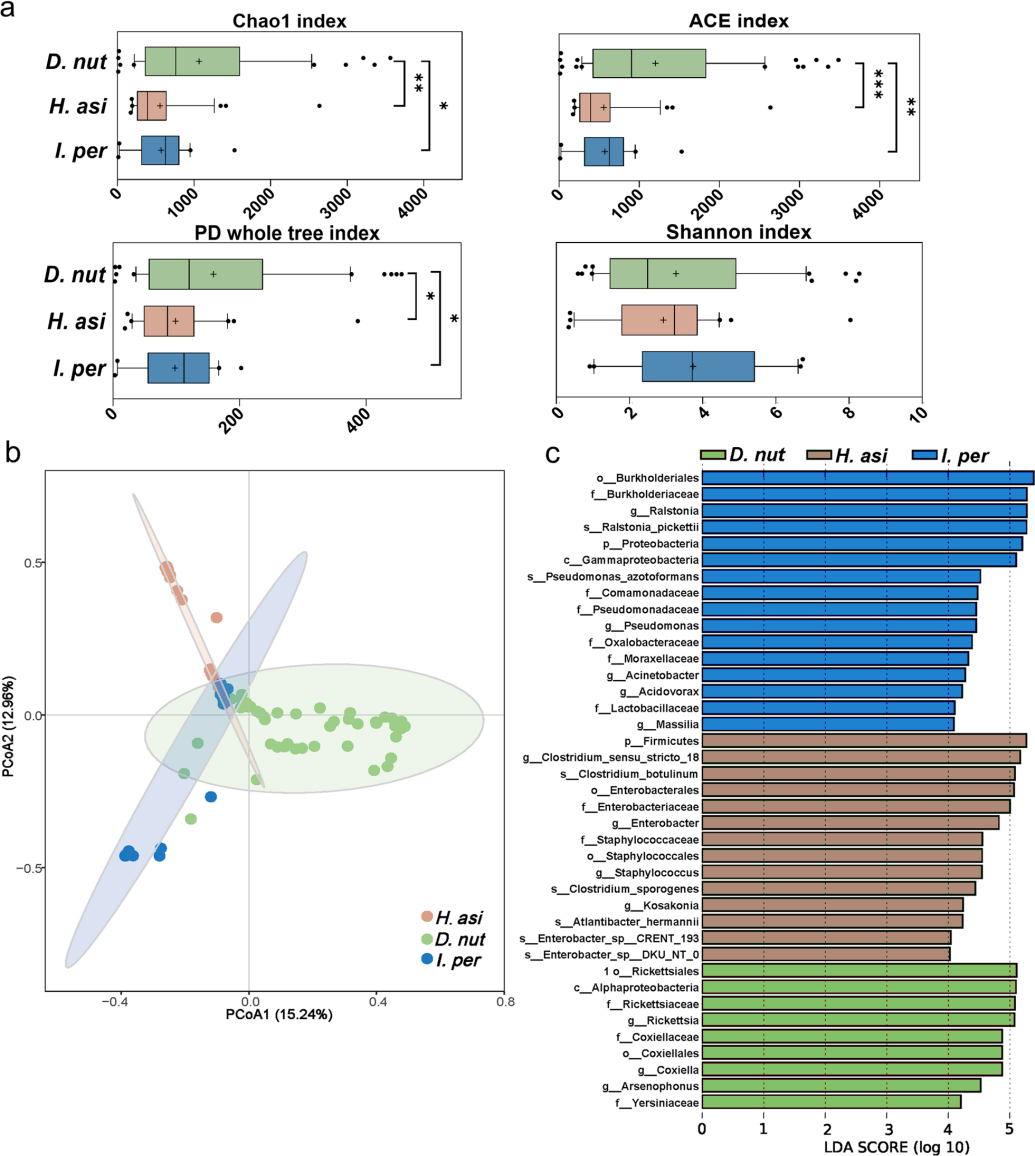
Microbial diversity and variation among *D. nuttalli*, *H. asiaticum*, and *I. persulcatus*. **a** Alpha diversity among *D. nuttalli*, *H. asiaticum*, and *I. persulcatus*. **b** PCoA plot based on Bray-Curtis distances, depicting the microbial community variation among ticks from different species, using their relative abundance data. **c** Cladogram of microbial communities among different tick species by LEfSe analysis. The threshold value is 4. *P*-values < 0.05 are considered significant. The line inside each box represents the median value, and "+" represents the mean value. Outliers are shown as dots; t-tests were performed for each pairwise comparison. *P < 0.05, **P < 0.01, ***P < 0.001

The alpha diversity of *D. nuttalli* was significantly higher than those of *H. asiaticum* and *I. persulcatus*, as demonstrated by the Chao, ACE, and PD whole tree index (*P* < 0.05) (Fig. 2a). Among three tick species, *D. nuttalli* was found to have the most bacterial genera. Notably, *D. nuttalli* displayed the highest number of unique bacterial genera, accounting for 22.6% (376/1661) of the total identified microbial genera among the three tick species. In contrast, there was no significant difference in alpha diversity between *H. asiaticum* and *I. persulcatus*.

The beta diversity analysis results showed distinct clusters among *D. nuttalli*, *H. asiaticum*, and *I. persulcatus* (Fig. 2b). The analysis revealed that some samples of *H. asiaticum* and *I. persulcatus* clustered closely with *D. nuttalli*. The ANOSIM analysis confirmed that there were significant differences among *H. asiaticum*, *D. nuttalli*, and *I. persulcatus* (*R* = 0.463,* P* = 0.001 < 0.05) (Fig. S2a). LEfSe analysis revealed significant differences among *Ralstonia*, *Pseudomonas*, *Acinetobacter*, *Acidovorax*, *Massilia*, *Clostridium *sensu stricto* 18*, *Enterobacter*, *Staphylococcus*, *Kosakonia*, *Acinetobacter*, *Rickettsia*, and *Coxiella* (Fig. 2c).

### Microbial variation under different geographical origins

**Fig. 3:**
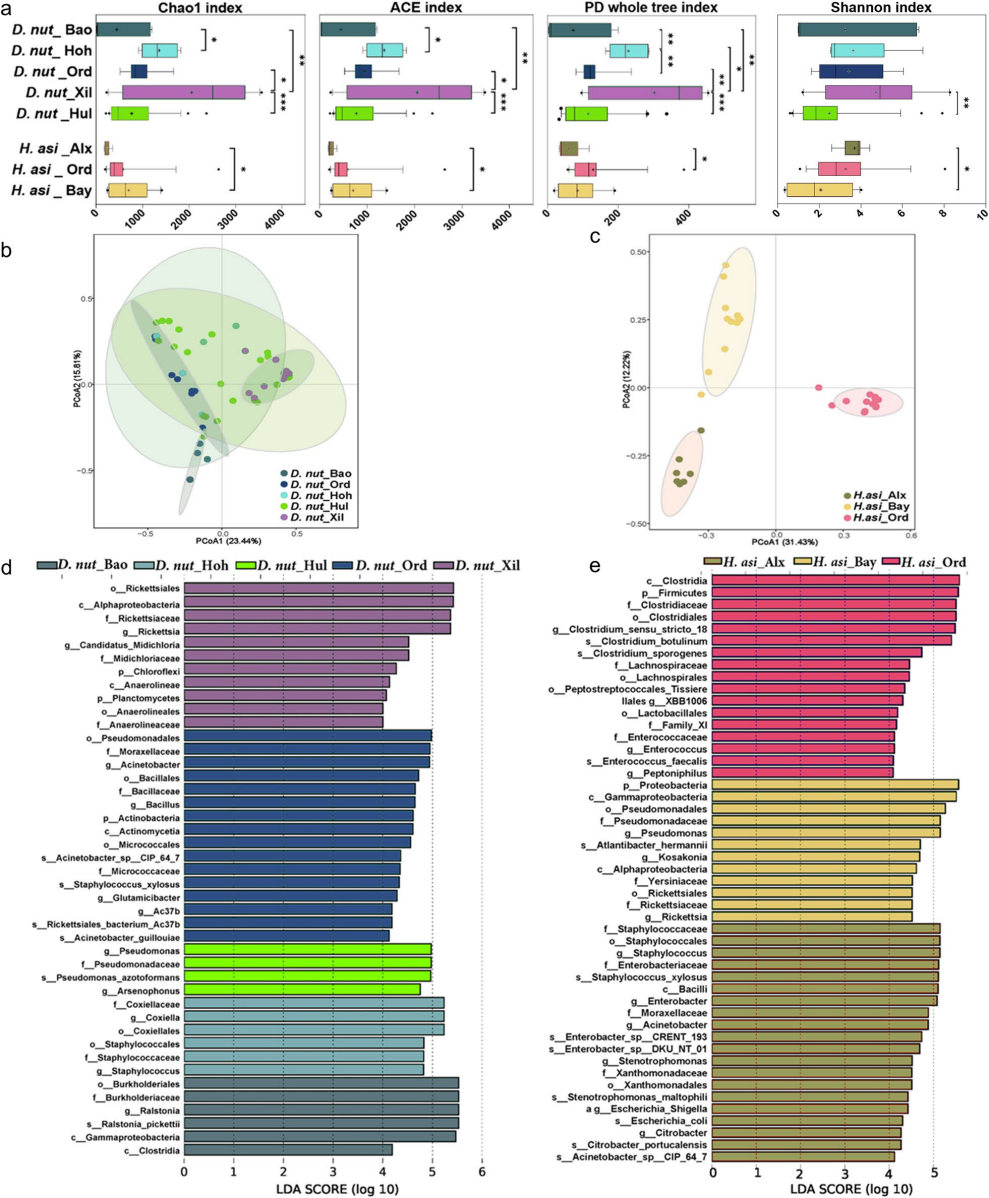
Microbial variation under different geographical origins. **a** Alpha diversity of *D. nuttalli* and *I. persulcatus* from different geographical locations. **b**, **c** PCoA plot based on Bray-Curtis distances, depicting the microbial community variation between *D. nuttalli* and *H. asiaticum* from geographical origins, using their relative abundance data. **d**, **e** Cladogram of microbial communities among *D. nuttalli* and *H. asiaticum* from geographical origins by LEfSe analysis. The line inside each box represents the median value, and "+" represents the mean value. Outliers are shown as dots; *t*-tests were performed for each pairwise comparison. *P < 0.05, **P < 0.01, ***P < 0.001

The alpha diversity of the microbial community in *D. nuttalli* and *H. asiaticum* varied significantly across different geographical locations. Compared to *D. nuttalli* from other regions (Baotou, Ordos, Hohhot, and Hulun Buir), the *D. nuttalli* from Xilingol League (*D. nut*_Xil) exhibited the highest microbial community diversity. The PD whole tree and Shannon index revealed that the microbial community diversity between Hulun Buir's (*D. nut*_Hul) and Xilingol League's *D. nuttalli* (*D. nut*_Xil) was the most pronounced (*P* < 0.001). Additionally, there was a difference in microbial alpha diversity among *H. asiaticum* from Alxa League, Ordos, and Bayan Nur (Fig. 3a).

PCoA analysis revealed that some *D. nuttalli* from Hohhot and Hulun Buir were dispersedly distributed, while *D. nuttalli* from other locations formed separate clusters (Fig. 3a, c). *H. asiaticum* from Alxa League, Ordos, and Bayan Nur formed separate clusters, respectively. ANOSIM analysis indicated significant differences with *R* = 0.498, *P* = 0.001 < 0.05 (Fig. S2b, c). LEfSe analysis was conducted to elucidate significant variations in microbial communities across different geographical origins. The outcomes are depicted through a histogram, accentuating the key taxa that differentiate among populations. For instance, *Rickettsia* was found to be significantly more abundant in *D. nuttalli* from Xilingol, while *H.* asiaticum of Ordos had more abundant *Clostridium *sensu stricto 18, *Enterococcusoccus*, and *Peptoniphilus* (Fig. 3d, e).

### Microbial variation in ticks by feeding status

**Fig. 4:**
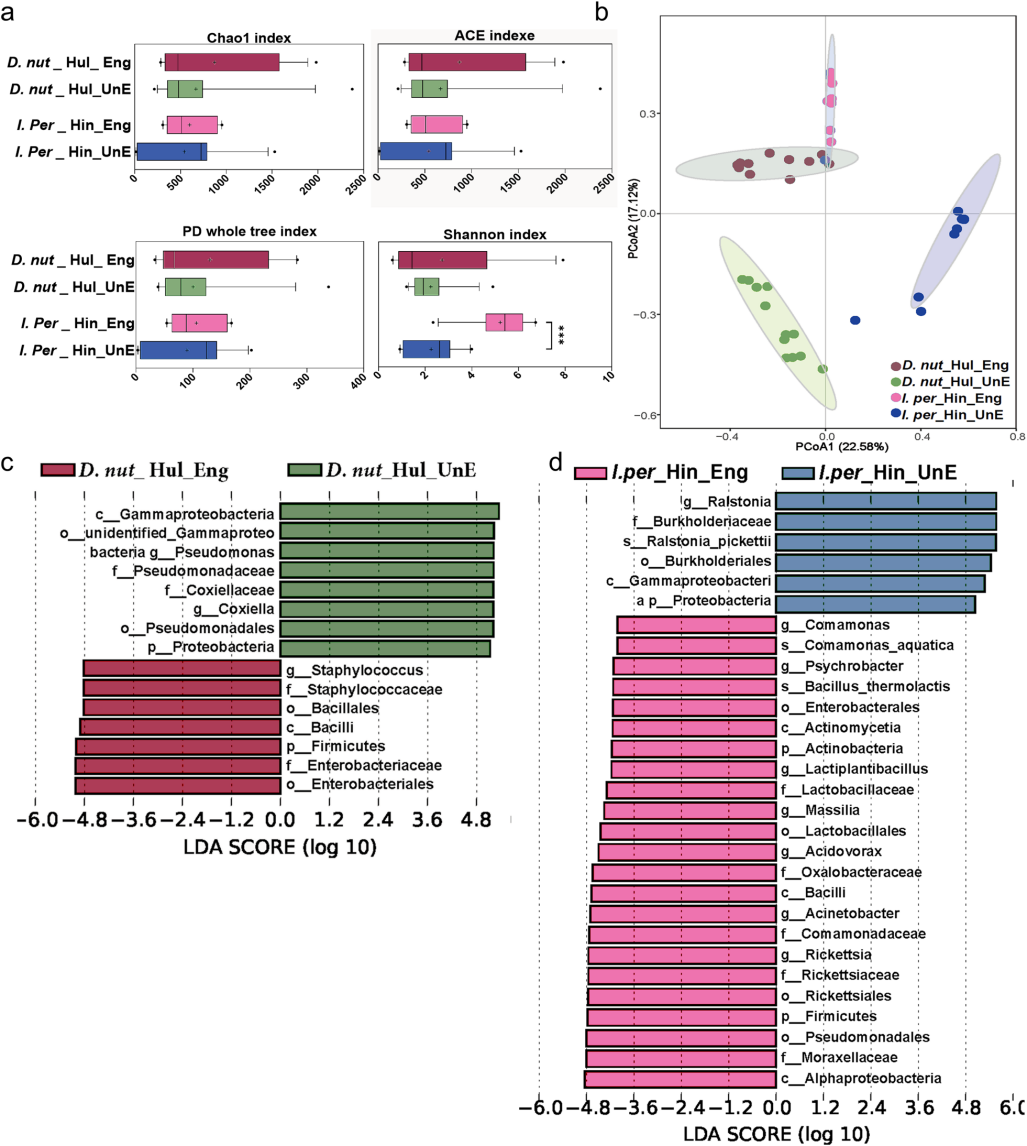
Microbial variation in ticks by feeding status. **a** Comparison of alpha diversity between engorged and unengorged *D. nuttalli*, engorged and unengorged *I. persulcatus*. **b** PCoA plot based on Bray-Curtis distances, depicting the microbial community variation among *D. nuttalli* and *I. persulcatus* with different feeding statuses. **c**, **d** Microbial communities of *D. nuttalli* and *I. persulcatus* under different feeding statuses by LEfSe analysis. The line inside each box represents the median value, and "+" represents the mean value. Outliers are shown as dots; *t*-tests were performed for each pairwise comparison. *P < 0.05, **P < 0.01, ***P < 0.001. The threshold value of 4, *P*-values < 0.05 are considered significant

Through alpha-diversity analysis, we found that engorged and unengorged *D. nuttalli* from Hulun Buir showed similar diversity levels, as shown in Fig. 4a; Chao1, ACE, and PD whole tree indices were not significantly different between engorged and unengorged *D. nuttalli* or engorged and unengorged *I. persulcatus*. However, there was a significant difference in the Shannon index between engorged and unengorged *I. persulcatus* (*P* < 0.05).

Microbial variation in ticks by feeding status differed significantly, as indicated by PCoA analysis (Fig. 4b). Furthermore, ANOSIM analysis confirmed significant differences among ticks with various feeding statuses (*P* < 0.05), highlighting the impact of feeding status on tick microbiota (Fig. S2d, e). LEfSe analysis showed lower abundances of *Pseudomonas* and *Coxiella* in engorged *D. nuttalli* compared to unengorged *D. nuttalli*, while the relative abundances of *Rickettsia*, *Lactiplantibacillus*, *Acinetobacter*, and *Massilia* in engorged *I. persulcatus* were higher than in unengorged *I. persulcatus* (Fig. 4c, d).

### Heterogeneity across different tick organs

**Fig. 5:**
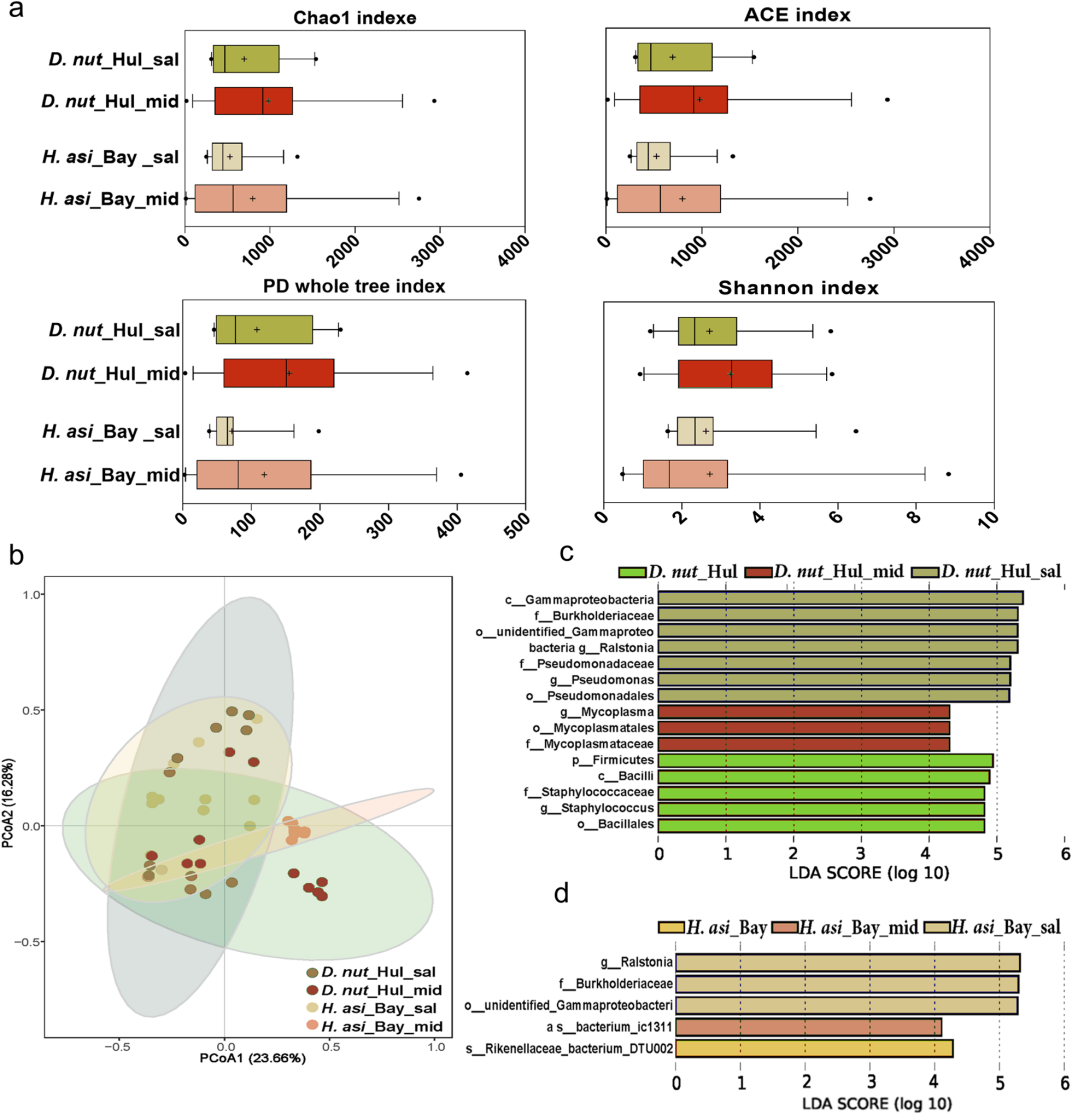
Microbial variation in the midgut and salivary glands of *H. asiaticum* and *D. nuttalli*. **a** Comparison of alpha diversity between the midgut and salivary glands of *D. nuttalli* and *H. asiaticum*. **b** PCoA plot based on Bray-Curtis distances, depicting the microbial community variation among the midgut and salivary glands of *H. asiaticum* and *D. nuttalli*. **c**, **d** Microbial communities of the salivary gland and midgut of *D. nuttalli* and *H. asiaticum* by LEfSe analysis. The line inside each box represents the median value, and "+" represents the mean value. Outliers are shown as dots. *t*-tests were performed for each pairwise comparison. *P < 0.05, **P < 0.01, ***P < 0.001. The threshold value of 4, *P*-values < 0.05 are considered significant

The microbial community diversities in the salivary glands and midguts of *H. asiaticum* and *D. nuttalli* were similar, as indicated by alpha diversity (Fig. 5a). The common microbial genera in the salivary glands of *D. nuttalli* and *H. asiaticum* constituted 41.71% (282/676) of the total genera. Likewise, the shared microbial genera in the midgut of *D. nuttalli* and *H. asiaticum* accounted for 46.93% (466/993). These results indicate a significant overlap in bacterial community composition within the same organ across different tick species, suggesting that organ-specific environments may select particular microbial assemblages.

The PCoA analysis revealed a similar bacterial community composition in the salivary glands of *H. asiaticum* and *D. nuttalli*. This finding was supported by the ANOSIM analysis, which produced a *P*-value of 0.312, indicating no significant difference between the salivary gland microbiomes of the two tick species (Fig. 5b). In contrast, a comparison of the midgut bacterial communities of *H. asiaticum* and *D. nuttalli* showed that they clustered separately, with a significant *P*-value of 0.001, highlighting distinct differences between the species (Fig. 5c, d). Further analysis within the same species showed that the salivary glands and midgut of *H. asiaticum* formed distinct clusters, as demonstrated by the PCoA analysis (ANOSIM: *R* = 0.581, *P* = 0.001). For *D. nuttalli*, the ANOSIM analysis also indicated a significant differences between the salivary glands and midgut, with a *P*-value < 0.05 (Fig. S2f, g). To further explore organ-specific microbial compositions, a comparative analysis was conducted, revealing that the family Burkholderiaceae was significantly more abundant in the salivary glands than in the midgut of both *D. nuttalli* and *H. asiaticum* (Fig. 5c, d).

### Potentially tick-borne pathogenic bacteria

**Fig. 6:**
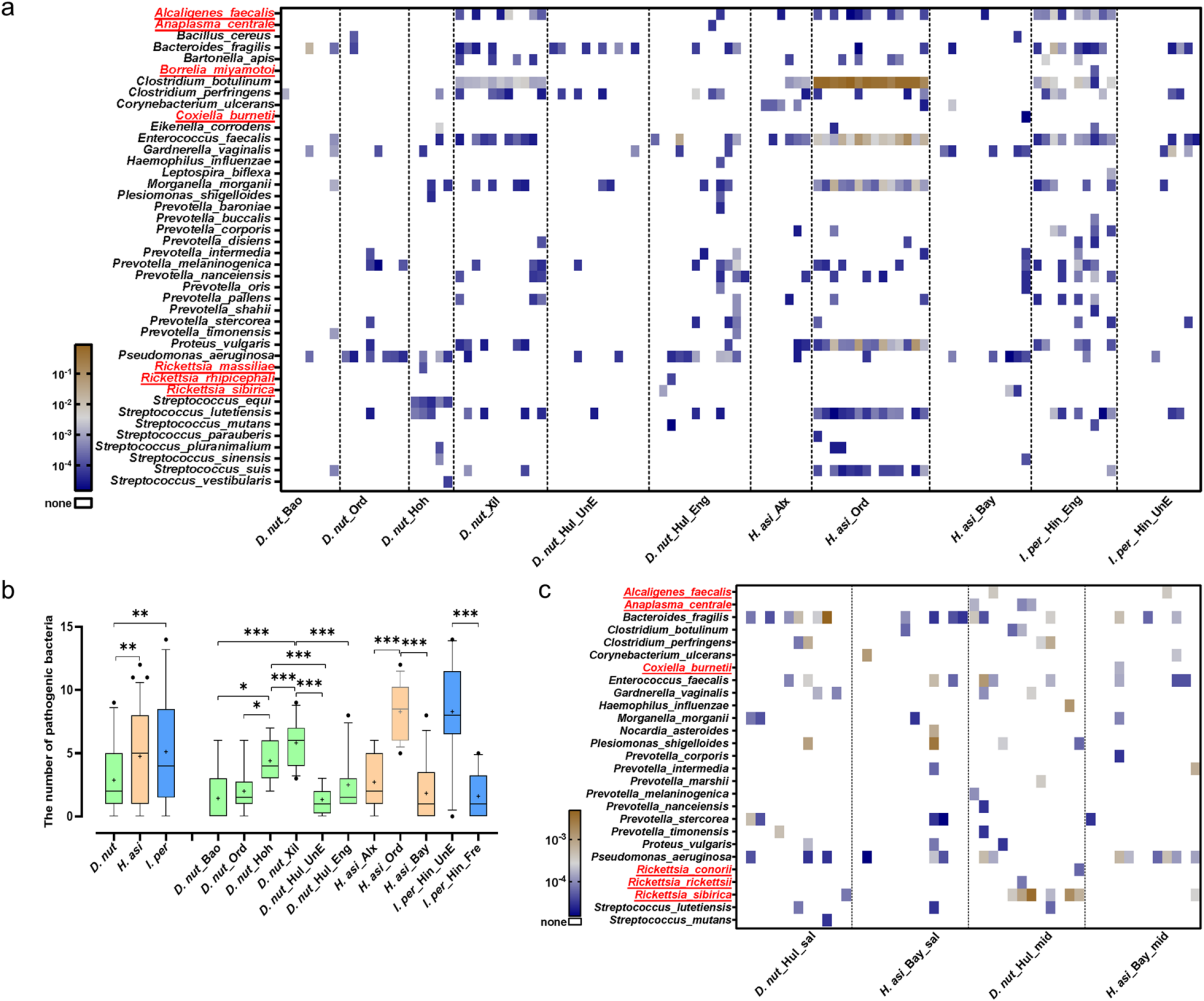
Characteristics of pathogenic bacteria contained in ticks. **a** Heat map of the relative abundance of pathogens detected in *D. nuttalli*, *H. asiaticum*, and *I. persulcatus* from different groups. **b** The number of pathogenic bacteria carried by *D. nuttalli*, *H. asiaticum*, and *I. persulcatus* from different geographical locations. **c** Heat map of the relative abundance of pathogenic bacteria detected in the midgut and salivary glands of *D. nuttalli* and *H. asiaticum*. Pathogenic bacteria marked in red and underlined are tick-borne pathogens known to cause human disease in China

Identification of tick-borne pathogens or pathogens ticks can carry is essential for understanding the risk of tick-borne diseases and developing strategies for their prevention and control. First, we identified a total of 46 known human pathogenic bacteria in all samples by comparing them with the 16S pathogen database (Fig. 6a; Table S3). We identified nine known tick-borne pathogens in all samples, including *Rickettsia conorii*, *R. massiliae*, *R. rhipicephali*, *R. rickettsii*, *R. sibirica*, *Acinetobacter faecalis*, *Anaplasma centrale*, *Borrelia miyamotoi* and *Coxiella burnetii* (Fig. 6a).

Significant differences exist in the number of pathogenic bacteria carried by different tick species. Notably, *D. nuttalli* harbors the most diverse array of tick-borne pathogens, including: *R. massiliae*, *R. conorii*, *R. rhipicephali*, *R. rickettsii*, and *A. centrale*. In contrast, *C. burnetii* was exclusively detected in *H. asiaticum*, and *B. miyamotoi* was found only in *I. persulcatus* (Fig. 6b). The number of pathogenic bacteria in *D. nuttalli* from the Xilingol League was higher than in other geographical locations. Similarly, *H. asiaticum* from Ordos had a higher abundance of pathogenic bacteria compared to Alxa League and Bayan Nur. The number of pathogenic bacteria carried by unengorged *I. persulcatus* is significantly greater than that in unengorged *I. persulcatus*. Most pathogenic bacteria were found in the salivary glands and midgut of ticks, suggesting these organs are critical sites for pathogen acquisition and transmission (Fig. 6c). Additionally, *D. nuttalli*'s salivary glands and midgut carried more pathogens compared to *H. asiaticum*, indicating a higher transmission risk associated with *D. nuttalli*.

## Discussion

Ticks are widely distributed vectors of pathogens, attracting significant public health attention due to the emergence and re-emergence of infectious diseases they cause [44, 45]. Previous research on tick bacteria, especially those using partial 16S rRNA gene sequencing (e.g. V3–V4 regions), has predominantly identified bacteria at the genus level. There have been comparatively few studies capable of determining bacteria at the species level. In this study, we collected dominant tick species, including *D. nuttalli*, *H. asiaticum*, and *I. persulcatus*, from various geographic locations across western, central, and eastern Inner Mongolia, China. Utilizing third-generation sequencing technologies, we obtained the full-length sequence of the 16S rRNA gene, allowing for a more accurate analysis of the composition and diversity of tick bacterial communities. The identified OTUs were classified into 142 phyla, 278 classes, 503 orders, 752 families, 1661 genera, and 2708 species, significantly enhancing the degree of microbial discrimination.

Proteobacteria were the most abundant bacteria in all three tick species, followed by Firmicutes and Bacteroidetes, consistent with previous studies on tick bacterial community composition [46–48]. This indicates a global similarity at the phylum level of tick bacterial community composition. At the genus level, *D. nuttalli*'s bacterial community composition varied significantly across different locations in Inner Mongolia. Compared to *D. nuttalli* and *H. asiaticum*, *I. persulcatus* had a relatively less complex microbial community, with *Ralstonia* and *Pseudomonas* accounting for > 52.11% of the total abundance. Studies have shown that a high abundance of *Pseudomonas* was also detected in *I. persulcatus* in Northeast China, but the relative abundance of *Ralstonia* was lower, likely due to geographical differences [49]. *Rickettsia*, serving as both symbionts and potential pathogens, including *R. massiliae*, *R. conorii*, *R. rhipicephali*, and *R. rickettsii* identified in this study, exhibited high relative abundances in *D. nuttalli* and *H. asiaticum* ticks, while demonstrating the lowest relative abundance in *I. persulcatus*. *Enterobacter* and *Clostridium *sensu stricto 18 were more prevalent in *H. asiaticum*'s microbiome. Ralstonia was most abundant in *D. nuttalli* from Baotou, while *Coxiella* predominated in *D. nuttalli* from Hohhot, Ordos, Xilingol League, and Hulun Buir. These findings suggest a complex relationship between ticks and microbial communities, influenced not only by tick species but also by geographical location [50, 51].

The host's blood plays a crucial role in the embryonic development and molting of ticks, and the blood-feeding state may influence the tick's bacterial community composition [52]. Our study revealed significant differences in microbial communities between engorged and unengorged ticks. The changes in microbial abundance during different feeding states vary among tick species. For example, the relative abundance of *Coxiella* and *Rickettsia* in *Haemaphysalis flava* changes with feeding status [53], consistent with our findings. While *Coxiella* remains the most abundant in both engorged and unengorged *H. longicornis*, its relative abundance decreases after blood feeding [54]. Notably, the observed decrease in relative abundance may be associated with an increase in alpha diversity and more species being sequenced. Engorged ticks can acquire microorganisms through the ingestion of blood, thereby enhancing the diversity of bacterial species. Nonetheless, as the ingested blood is metabolized, these microorganisms suffer from concomitant loss. To mitigate this reduction, ticks could supplement their microbiome with bacteria sourced from their surrounding environment [55, 56]. This study revealed significant disparities in the composition and diversity of bacterial communities within *D. nuttalli* and *H. asiaticum* ticks from varying geographical locations despite being at the same stage of blood feeding. These findings suggest that geographical factors potentially influence the bacterial community composition more than the feeding status of the ticks. However, more attention should be paid to the potential influence of geographical variation, which may be affected by factors such as ethanol washing and environmental contamination.

Ticks acquire bacteria that must pass through the gut to colonize, and saliva produced by the salivary glands is required for transmission [57]. Our results demonstrated that the bacterial community composition in the salivary glands of *D. nuttalli* and *H. asiaticum* was similar, as shown by clustering in beta diversity analysis. However, the bacterial community composition of the midguts of *D. nuttalli* and *H. asiaticum* differed significantly. Within the same tick species, the bacterial community composition of the saliva and midgut also showed significant differences, with similar organ-specific variations observed in *D. silvarum* [58]. The salivary glands of *D. nuttalli* and *H. asiaticum* exhibited a higher relative abundance of *Burkholderiaceae Ralstonia* and unidentified *Gammaproteobacteria*, which were the dominant species in the salivary glands. These microorganisms were not as abundant in the midgut, likely because of species-specific differences in tick biology.

Our research also identified a variety of pathogens in the dominant tick species in Inner Mongolia, including several common tick-borne pathogens such as *B. miyamotoi*, *R. conorii*, *R. massiliae*, *R. rhipicephali*, *R. rickettsii*, *R. sibirica*, *A. faecalis*, *A. centrale*, and *C. burnetii* [59–62]. Notably, *D. nuttalli* harbored more tick-borne pathogens than *H. asiaticum* and *I. persulcatus*, suggesting that it plays a more significant role in the transmission of infectious agents. The prevalence of pathogenic bacteria in *D. nuttalli* from the Xilingol League was higher than in other regions. Similarly, *H. asiaticum* from Ordos exhibited a greater abundance of pathogenic bacteria compared to those from the Alxa League and Bayan Nur. Moreover, the number of pathogenic bacteria detected in the midgut was higher than in the salivary glands. These findings indicate that tick species, geographic location, and organ type influence the distribution and prevalence of these pathogens. Previous studies have reported that feeding behavior and host preference could contribute to this increased pathogen load [63, 64], as these factors may determine a tick's exposure to various pathogens and its ability to transmit them [65–67]. Additionally, we discovered multiple potential tick-borne pathogens associated with urinary tract and gastrointestinal infections, which can cause severe, life-threatening conditions [68–70]. Some of these pathogens have been detected in *Rhipicephalus* spp. and *Amblyomma* spp. (not identified to species level), though there have been no reports of direct infection from tick bites [61]. These findings underscore the diverse pathogenic bacteria carried by ticks in Inner Mongolia. However, further analysis of tick-borne pathogens and vector competence is needed to assess the actual transmission risk accurately.

While this study characterized ticks from different locations in Inner Mongolia to assess their microbial diversity, there were limitations. First, using ethanol to wash ticks and the lack of a blank control hindered accurate evaluation of microbial diversity. Second, *I. persulcatus* was collected from a single location, making it impossible to assess geographical influence on its bacterial community. However, the bacterial community composition and diversity of ticks in Inner Mongolia differed from those in Heilongjiang and Jilin Provinces, suggesting geographic influence [49]. Third, no other tick-associated pathogens were detected, contrasting with previous studies reporting *Ehrlichia* sp. and *Anaplasma phagocytophilum* in *I. persulcatus* and *D. nuttalli* in northeastern Inner Mongolia [34]. This discrepancy could be due to different sampling locations, smaller sample sizes, and detection limitations. Finally, while the amount of blood feeding relates to bacterial community composition, the associations between bacterial community composition and blood hosts cannot be well explained because of a lack of ticks from different hosts in the same location [71–73]. Our results indicate that the microbial diversity of ticks is influenced by geographic location, blood-feeding amount, and organ specificity. However, it remains unclear which factor plays a pivotal role in determining microbial diversity. Future studies should expand the geographic locations and host types of tick samples to explore the interactions between geographic location, host types, and other environmental factors.

## Conclusions

This study characterized the microbial communities of the three dominant ticks, *D. nuttalli*, *H. asiaticum*, and *I. persulcatus*, in different geographical locations in Inner Mongolia. Our results indicated that the microbial community of *D. nuttalli* differed significantly from that of *H. asiaticum* and *I. persulcatus*. It is important to note that the tick microbiome is regulated by the tick’s geographical location, feeding status, and specific organs. Moreover, ticks carry a variety of pathogenic bacteria in Inner Mongolia, and new tick-borne pathogens may emerge. Hence, it is crucial to enhance tick and tick-borne disease surveillance and monitoring in the region for early detection, understanding ecological factors, assessing disease burden, and implementing prevention and control measures.

## Supplementary Information

Supplementary Material 1: Table S1. The taxonomy and relative abundance in each sample.

Supplementary Material 2: Table S2. The relative abundance of the top 10 phyla in all samples.

Supplementary Material 3: Table S3. The 46 microorganisms known to be associated with human disease

Supplementary Material 4: Figure S1. The relative abundance of microbial taxa among different groups.

Supplementary Material 5: Figure S2. ANOSIM analysis of the differences between different species (a), geographical locations (b, c), feeding states (d, e), and organs (f, g, h, i).

## Data Availability

No datasets were generated or analysed during the current study.
