# Supplementary figures and images for: Deciphering the microbial communities in ticks of Inner Mongolia: ecological determinants and pathogen profiles
Source: Parasit Vectors. 2024 Nov 4;17:448. doi: 10.1186/s13071-024-06512-1 (PMC11533347; doi:10.1186/s13071-024-06512-1)

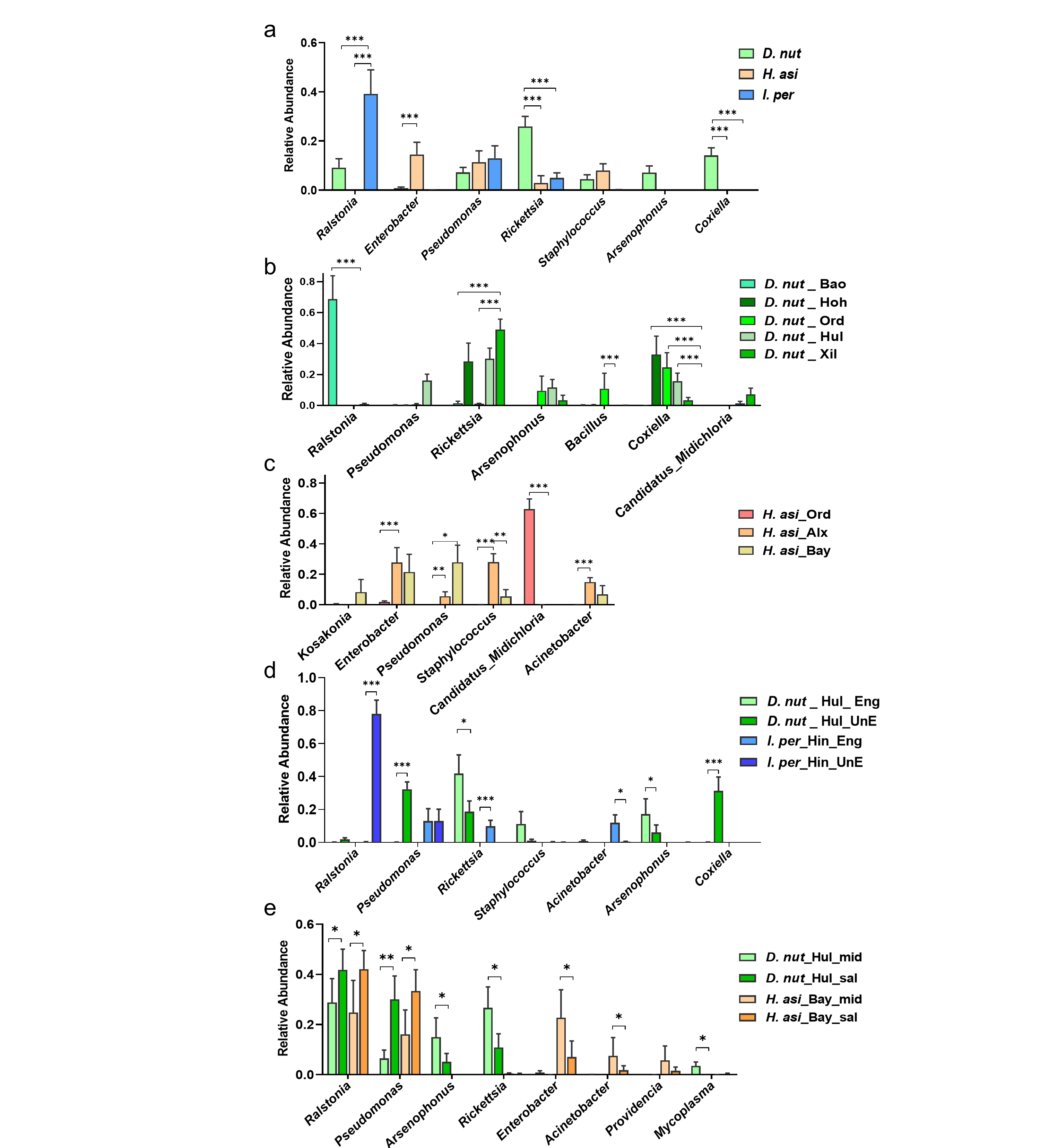

Supplement: Supplementary file 4 — Supplementary Material 4: Figure S1. The relative abundance of microbial taxa among different groups. [file 13071_2024_6512_MOESM4_ESM.tif]

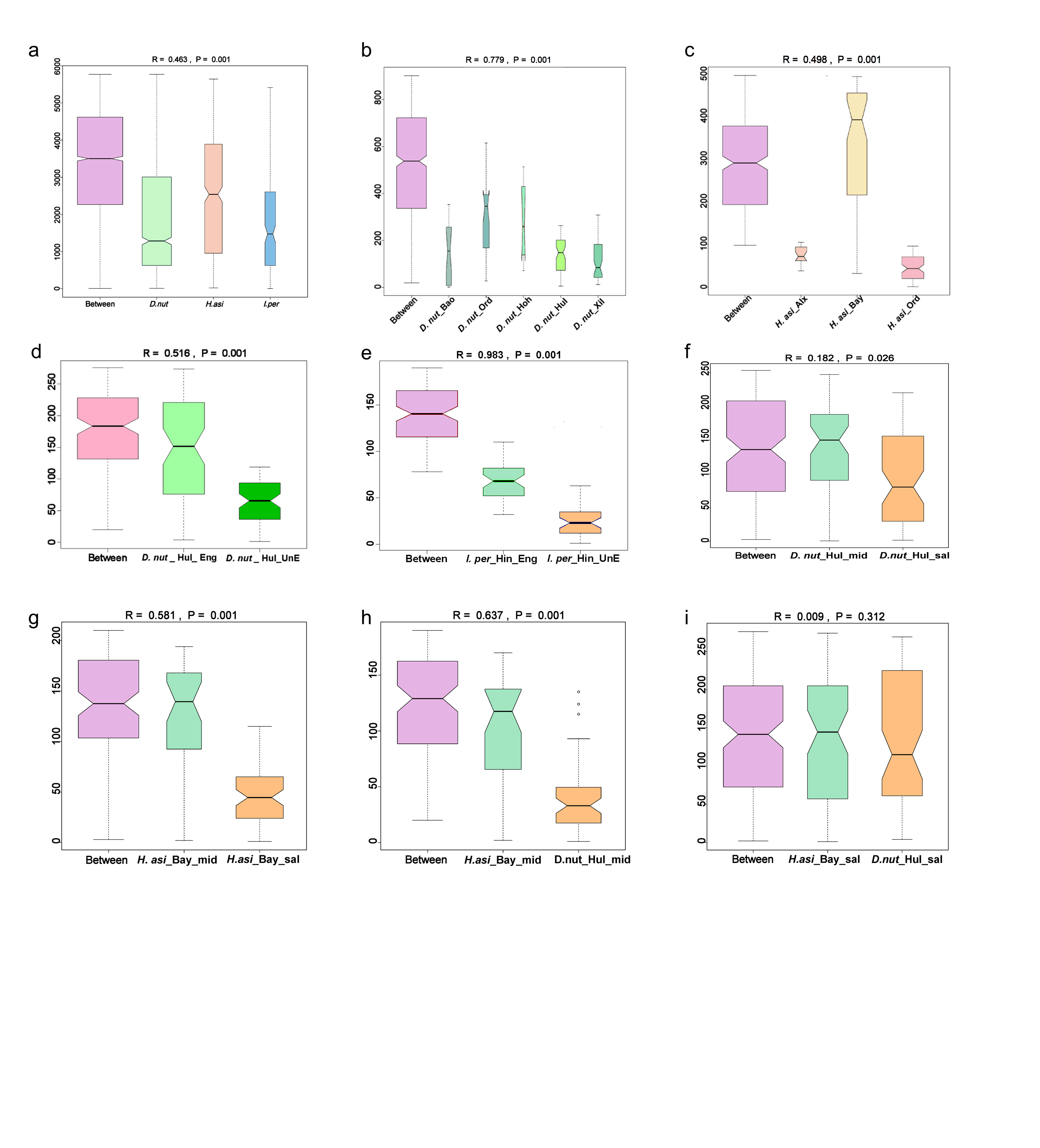

Supplement: Supplementary file 5 — Supplementary Material 5: Figure S2. ANOSIM analysis of the differences between different species (a), geographical locations (b, c), feeding states (d, e), and organs (f, g, h, i). [file 13071_2024_6512_MOESM5_ESM.tif]
